# Primary Vesicoureteral reflux and chronic kidney disease in pediatric population. What we have learnt?

**DOI:** 10.1590/S1677-5538.IBJU.2020.02.02

**Published:** 2020-01-10

**Authors:** Veridiana Andrioli, Rodrigo Regacini, Wilson Aguiar

**Affiliations:** 1 Departamento de Cirurgia Escola Paulista de Medicina Universidade Federal de São Paulo São PauloSP Brasil Divisão de Urologia e Departamento de Cirurgia, Escola Paulista de Medicina, Universidade Federal de São Paulo – UNIFESP, São Paulo, SP, Brasil;; 2 Divisão de Radiologia Pediátrica Escola Paulista de Medicina Universidade Federal de São Paulo São PauloSP Brasil Departamento de Imagem Médica e Divisão de Radiologia Pediátrica, Escola Paulista de Medicina, Universidade Federal de São Paulo – UNIFESP, São Paulo, SP, Brasil

## INTRODUCTION

Vesicoureteral reflux (VUR) can be defined in a simplistic way as a retrograde flow of urine from the bladder to the upper urinary tract, affecting approximately 30% for children with urinary tract infection (UTI) and 17% without infection (1). However, when including only children that presented with UTI in the first year of life, this percentage now represents up to 70% (2).

It is worth to mention that, asymptomatic infants followed post-natal for pre natal hydronephrosis who had resolved or downgraded the renal dilatation previously identified, a prevalence of 15% of VUR still can be found (3).

The medical interest of VUR contemplates famous public figures from the past as Galeno and DaVinci, however, was only in the 1970’s with Ransley and Risdon (4) that the relationship amongst urinary tract infection, vesicoureteric reflux, and renal scar were determined.

Interesting, the classification following grades based on voiding cystourethrography (VCUG) was only established in 1985 (5). Since the very beginning, special attention was turned to dilated grade VUR (Grades 3 to 5), mainly in males.

Back in 1965, Bialestock (6) postulated an evident relation between pyelonephritis and VUR. Marra et al. (1994) (7) and later Swerkersson S. et al. (2007) (8) showed a direct relationship between males, high-grade VUR and renal dysplasia. Their founds were reinforced in 2010 by Craig and Rushton (9).

With the advance of antenatal images and consequently early suspicion of renal / ureteric abnormalities and hydronephrosis, newborns are deeply investigated following well-established protocols (10) and, when indicated, promptly initiated on antibiotic prophylaxis or even, surgical intervention.

In terms of intervention for VUR patients, which will be better revised later in this article, we changed from invasive to conservative management mainly after 1997 following Yeung and colleague’s publication (11).

Curiously, in terms of high-grade primary VUR, the early diagnosis, and precocious intervention seem not to be capable to prevent children from developing renal scars and even worse, chronic kidney disease (CKD) including renal failure.

Numbers obtained in 2006 from The North American Pediatric Renal Trials and Collaborative Studies (NAPRTCS) (12) and The Italian Kid Project (2004) (13) showed that, amongst pediatric population, respectively 5.2% and 25% of patients on renal replacement therapy (peritoneal or hemodialysis and/ or renal transplantation) were due to reflux nephropathy or past medical history of VUR.

Then, VUR is not only back-flow urine. At least, it does not seem to be in patients with reflux nephropathy, term established in 1973 by Bailey (14). In this cohort, besides VUR follow-up, special attention should be taken to the patient’ somatic growth, hypertension, UTIs as well as kidney’s aspect on imagens, including signs of dysplasia, grade of hydronephrosis and ureteric dilatation. All these characterizes must be seen as a single component added to genetic predisposition and socioeconomic condition, among others (15).

In this article, we intend to review the most significant literature in terms of primary VUR and CKD.

We will also explore some definitions and imaging resources that might help physicians to better understand this particular group of VUR patients.

## HISTOLOGICAL ASPECTS

First of all, it is important to understand some concepts and definitions regarding the kidney’s histological aspect, and primordially determine differences between an acquired scar from a dysplastic/ hypoplastic kidney.

The term renal dysplasia reports to an anomalous differentiation of the renal parenchyma which is a consequence of abnormal interaction of the ureteric bud. One mechanism that can possibly interfere on the ureteric vesical junction (UVJ) balance may be an ongoing mechanical high-pressure of sterile fetal VUR, and can be associated to a temporarily abnormal bladder behavior with hypertonicity and uncoordinated voiding happening during gestation (mainly in male patients) (6). These changes seem to drive to a peculiar and definitive renal parenchyma tissue of rudimentary nephronic structures (16, 17).

Craig and Rushton (9), considered that “Congenital renal impairment” would be a better term to describe these antenatal compromised kidneys.

On the other hand, a hypoplastic kidney is the one that has a reduction of the total mass of glomerulus and tends to be a globally smaller kidney (17).

On the opposite, renal scar is acquired post-natal following an acute inflammatory reaction from bacterial infection (8). In this condition, females with dilated reflux had a greater chance to develop acute pyelonephritis and, consequently, renal scarring. Here, different from boys with congenital changes, low doses of prophylactic antibiotics seem to reduce the risk of renal scarring (18).

Long term consequences for patients with renal scars are well known and include hypertension, proteinuria, compromising somatic growth and renal failure.

## WHAT SHOULD WE LOOK ON IMAGES?

Urinary tract dilatation is the first clue of VUR seen in the Ultra sound (US) , which is a non-invasive, without exposure to ionizing radiation and available at the majority of the centers. It provides high-resolution anatomical images of renal parenchyma, calyx, pelvis, and bladder. Ureters and urethra are only partially visualized.

First of all, imaging methods can be categorized into those that provide anatomical detail (US, VCUG and urography magnetic resonance imaging (MRI-urography) , those that provide functional information (DMSA nuclear scintigraphy, or both (Functional-MRI (fMRI) and contrast-enhanced US (CE-US) ) (19).

In terms of US, other than evaluating the urine collection system, it can provide anatomical details of renal parenchyma that are critically important for the diagnosis and follow-up of vesical reflux nephropathy. Progression to chronicity can be noted in US in kidneys followed longitudinally. We can identify in follow up images reduction of the parenchyma thickening associated to an increase on cortical echogenicity.

The kidneys may present at US irregular characteristics as renal asymmetry, distorted papillae with caliectasis, pseudo nodular areas due to segmental hypertrophy, corticalization with parenchymal loss, cystic dysplasia and parenchymal scars. These last ones can be suspected in kidneys previously affected by pyelonephritis episodes.

It is worth to mention the important difference between a multicystic dysplastic kidney (MCDK) and a kidney with cystic dysplasia. Mercado-Deane et al (2002) describes this difference as: “A dysplastic kidneys usually retain a reniform shape and have more abundant parenchyma than classic MCDK The kidneys are normal to small in size with highly echogenic cortex, loss of corticomedullary differentiation, and scattered cysts that are smaller than those commonly seen with MCDK” (20).

Another interesting finding is a radial and regular arrangement of vessels on Doppler US done on top of areas of pseudo nodularization (21-23). Doppler tracing may also show a reduction of the wave profile amplitude due to glomerular atrophy secondary to renal mass loss (fibrotic replacement), leading to transmural renal pression with reduction of vascular capacitance (24).

DMSA scintigraphy is the gold standard for quantification of the renal parenchyma functioning (25), and, can be done in an acute episode of pyelonephritis or later to confirm established scars, although the imaging findings can be similar, it is necessary to evaluate images in comparisons from different times for definition. DMSA replaced the intravenous urogram (IVP) reducing significantly the time and radiation exposure, however, it does not provide anatomical details.

MRI-urography (26-28) is a method capable of providing high-resolution anatomical details of the entire urinary system. Its main indication includes complex genitourinary malformations, in which the detailed anatomy of the entire collecting system in three-dimensional or panoramic images is desired, being the best method for characterizing the ureters (especially useful in the evaluation of ectopic ureter).

MR urography is able to distinguish areas of acute infection from initial scarring by the different enhancement patterns of these two processes, which is an advantage over DMSA scanning in the evaluation of pyelonephritis and renal scarring at any time.

Although MRI does not use ionizing radiation, its main downside that broadly limits its use are examination time (on average 40 minutes) and the need for general anesthesia in children under 5 years. For this reason, the initial investigation with US and VCUG remains the most appropriate.

MRI also allows adding functional assessment using applicable programs and protocols (fMRI). With this, it is possible to indirectly obtain glomerular filtration rates of each kidney, renal parenchyma perfusion curves, and collection system excretion curves. It is especially useful in cases of ureteric hydronephrosis where scintigraphy is inconclusive and in postoperative cases where large residual system dilatation still remains.

Regards iMRVC for evaluation of VUR it is similar to a conventional VCUG, using instead, gadolinium into the urinary bladder, with the advantage of providing greater anatomical details and continuous surveillance for VUR during the entire filling, waiting, and voiding phases of the study.

Unfortunately, given the limitations of MRI in young children, this technique presents itself only as a possible alternative when other tests cannot be performed or are not conclusive.

## WHAT IS THE PERCENTAGE OF CHILDREN WITH END STAGE RENAL DISEASE RELATED TO PRIMARY VUR?

Define an accurate incidence of VUR is difficult. We already saw that it can be underestimated, mainly for these patients that never had a febrile UTI, or even for the ones that could have a past history of VUR, however, when presented with complications including renal scars could have it spontaneously resolved (11, 29).

Numbers from ANZDATA (The Australia and New Zealand Dialysis and Transplant Registry) from a retrospective study from 1971 to 1991 showed that 6.1% of men and 9.1% of women that were listed on renal transplant program had a reported past history of nephropathy VUR. This percentage goes up to 21% and 25% of boys and girls, respectively when looking only for patients younger than 16 years of age (30).

When accessing data from North America, numbers from the NAPRTCS (North American Pediatric Renal Trials and Collaborative Studies) there is an estimative of 3.5% to 5.2% of the children in renal replacement therapy because of VUR nephropathy (12).

A Canadian report from 1995 describes that approximately 20-25% of the children younger than 15 years had a history of pyelonephritis and VUR. Around 5% of all ages listed had CKD due to reflux nephropathy (31). A similar number was found in Europe (32).

Most recent numbers from Canada from Canadian Organ Replacement Register Annual Report (33) now showed in a restricted population from 11 to 17 years, a total of 12 teenagers (3.7%) listed for renal transplant due to VUR.

Interestedly to mention is the high number of patients with CKD in Italy secondary to VUR insults. Numbers from the ITALKID project and reported on Marra’s, et al. paper from 2004, described that 25.7% of patients with end stage renal failure were secondary to primary high grade VUR (grades 4 and 5). Of these, 77.5% were male (34).

In a Brazilian study from 2006, Silva et al. had accessed 735 children charts with primary VUR. Of the 684 patients followed longitudinally, 21 had CKD. A total of 10 patients progressed end stage CKF. However, their most interesting found was the fact that before 1990 an amount of 5% of the patients had CKD after 10 years follow up, but, after 1990, only 2% advanced to renal replacement therapy (35).

## CAN WE PREDICT WHICH GROUP OF CHILDREN WITH PRIMARY VUR ARE IN RISK FOR CKD?

Bailey’s paper earlier mentioned (14) and reinforced in Ishikura et al. (36) publication’s, conclude that VUR alone might not be responsible for kidneys’ deterioration. Their reports showed that the presence of hypoplasia/ dysplasia in refluxing units has a stronger association with the reduction of the glomerular filtration rate (GFR) other than VUR alone. Ishikura’s paper includes as risk factors to progression to end-stage renal insufficiency: puberty, stages 4 and 5 of CKD (GFR of 15-30 mL/min. and <15 mL/min. for CKD stage 4 and 5 respectively) and heavy proteinuria, here defined as urinary protein/ creatinine ratio >2.

Different from others here mentioned, Ishikura includes also patients with secondary VUR, for example males with posterior urethral valve.

Another author that demonstrated the straight relation between urinary protein, VUR and CKD was Sépibus et al. in 2017 (37), showing a downgrade on GFR in patients with high-grade VUR and high volume of urinary albumin excretion. This is reinforced by previously published literature (38, 39) and can be probably due to induced tubular atrophy and progressive renal failure.

In a prospective evaluation, an Italian report from 2004 from Caione et al. found that a creatinine level higher than 0.6mg/L obtained before one year of age and bilateral high-grade VUR were the most significative risk factors for worsening on renal function. Boys with these characteristics were at 125x risk of developing CKD in the first 6.3 years of life (40).

Sjöström et al. (41) in 2009 conducted a research by observing high-grade VUR patients that deteriorated renal function over the years. Curiously, there was no difference found between genders, however, patients with antenatal diagnosis and bilateral renal involvement at birth were the ones with a worse prognosis. This was similar to Silva et al. (35) findings.

Sjöström et al. also declared that worsening on patients’ renal function was a rare event, however, there was a tendency to deterioration with the increasing degree of VUR and bladder dysfunction. This last one is highly associated with breakthroughs UTIs.

## DOES SURGICAL INTERVENTION CHANGE THE FUTURE OF THESE CHILDREN?

We are living in an era in which observation over intervention is preferred. This is a truth since 1992 after Koff and Campbell (42) showed that was safety observe kids with antenatal unilateral hydronephrosis.

In terms of VUR active intervention regarding renal protection, only few years after Koff’s publication, Bayley et al. (30) described that proteinuria and hypertension could be persistent or appear despite a successful surgical intervention for VUR patients.

Craig et al. in 2000 (43-45) in a before-after study observed what happened in terms of renal function protection after the introduction of active treatment for VUR (surgical intervention and/ or antibiotic prophylaxis). The authors observed that there was no reduction in numbers of patients that were listed for renal transplantation due to high-grade VUR nephropathy over the years despite any therapy. Patients that had significant deterioration on GFR were that one that already had an abnormal DMSA scan at the very begin. This led them to conclude that surgical intervention is not indicated with the intention of preventing long-term renal damage.

Wheeler et al. (46) in a meta-analysis of randomized controlled trials also did not find significative benefit of any interventional treatment over antibiotics on the prevention of UTI or renal damage. Their most impressive result was that to prevent one single episode of febrile UTI in VUR patients under prophylaxis would be necessary nine reimplantations. And, even more interesting was the fact that there was no reduction in the number of children that developed renal damage and post-operative UTI.

Also, the most recent Cochrane review on interventions for primary VUR (47) showed that antibiotic prophylaxis had insignificant difference to the risk of new/ continuous renal damage in patients followed with DMSA scans.

## CONCLUSIONS

High-grade VUR must be considered a chronic disease and patients should be followed closely.

Unfortunately, patients with antenatal changes confirmed post-natal to have dysplastic kidneys, mainly males with bilateral involvement and high grade VUR tend to have a worst prognosis in terms of CKD.

Surgery and antibiotic prophylaxis seem not to protect against this evolution to renal failure, however, avoiding new onset scars preventing recurrent febrile UTI and treating aggressively urinary protein loss and comorbidities confer some protection to the urinary tract function.
